# Perspectives on obesity imaging: [^18^F]2FNQ1P a specific 5-HT_6_ brain PET radiotracer

**DOI:** 10.1038/s41366-024-01644-x

**Published:** 2024-10-07

**Authors:** Pierre Courault, Sandrine Bouvard, Caroline Bouillot, Radu Bolbos, Waël Zeinyeh, Thibaut Iecker, François Liger, Thierry Billard, Luc Zimmer, Fabien Chauveau, Sophie Lancelot

**Affiliations:** 1 Lyon Neuroscience Research Center (CRNL), CNRS UMR5292, INSERM U1028, Université Lyon 1, Lyon, France; 2 Hospices Civils de Lyon (HCL), Lyon, France; 3 CERMEP-Imaging Platform, Groupement Hospitalier Est, Bron, France; 4 Institute of Chemistry and Biochemistry (ICBMS), Université de Lyon, CNRS, Villeurbanne, France; 5 National Institute for Nuclear Science and Technology (INSTN), CEA, Saclay, France

**Keywords:** Obesity, Obesity, Preclinical research, Rat

## Abstract

**Background:**

Estimates suggest that approximatively 25% of the world population will be overweight in 2025. Better understanding of the pathophysiology of obesity will help to develop future therapeutics. Serotonin subtype 6 receptors (5-HT_6_) have been shown to be critically involved in appetite reduction and weight loss. However, it is not known if the pathological cascade triggered by obesity modifies the density of 5-HT_6_ receptors in the brain.

**Methods:**

Influence of diet-induced obesity (DIO) in Wistar rats was explored using MRI (whole-body fat) and PET ([^18^F]2FNQ1P as a specific 5-HT_6_ radiotracer). The primary goal was to monitor the 5-HT_6_ receptor density before and after a 10-week diet (DIO group). The secondary goal was to compare 5-HT_6_ receptor densities between DIO group, Wistar control diet group, Zucker rats (with genetic obesity) and Zucker lean strain rats.

**Results:**

Wistar rats fed with high-fat diet showed higher body fat gain than Wistar control diet rats on MRI. [^18^F]2FNQ1P PET analysis highlighted significant clusters of voxels (located in hippocampus, striatum, cingulate, temporal cortex and brainstem) with increased binding after high-fat diet (*p* < 0.05, FWE corrected).

**Conclusion:**

This study sheds a new light on the influence of high-fat diet on 5-HT_6_ receptors. This study also positions [^18^F]2FNQ1P PET as an innovative tool to explore neuronal consequences of obesity or eating disorder pathophysiology.

## Introduction

According to the World Health Organization (WHO), worldwide obesity rates have almost tripled since 1975. The WHO estimates that in 2016, the number of people with obesity reached over 650 million, or 13% of the adult population. Obesity is associated with higher risks of cardiovascular disease [1], diabetic renal or hepatic disorders [2], cancers [3] and mental disorders [4], leading to a higher risk of overall mortality [2]. European guidelines advocate reducing weight, body mass index and waist circumference, using three approaches: lifestyle modification, pharmacotherapy, and bariatric surgery [5]. The role of serotonin (5-HT) in eating behavior is clear and based on translational observations [6]. Stimulation of central 5-HT signaling emerged as a therapeutic target for obesity over a decade ago [7]. Unfortunately, the success of serotonergic drugs in the treatment of obesity has so far been limited by peripheral side effects due to the stimulation of serotonin receptors in peripheral tissues. For example, the 5-HT_2C_ receptor agonist, lorcaserin, has been shown to be effective in reducing food intake and body weight, but higher cancer risk and other complications led medical agencies (FDA and EMA) to withdraw it from the market [8, 9]. In this context, the recently discovered serotonin subtype 6 receptors (5-HT_6_) have emerged as a new potential target for obesity management. These receptors are almost exclusively located in the central nervous system: mainly in striatum, prefrontal cortex and hippocampus [10]. These receptors were shown to be involved in appetite regulation and weight variation [11]. Firstly, preclinical studies using 5-HT_6_ receptor-directed antisense oligodeoxynucleotides reported decrease in food consumption leading to lower body weight [12]. Secondly, treatment with 5-HT_6_ receptor antagonists resulted in similar effects [13–16]. Finally, a mouse model expressing a non-functional mutant of 5-HT_6_ receptor (C57BL/6) appeared to be resistant to diet-induced obesity (DIO) [17], and this was also observed in 5-HT_6_ receptor knock-out mice [18]. Several serotonergic drugs targeting 5-HT_6_ receptor have shown promising results with obesity, but more research is needed to determine whether this system can be safely targeted in this pathology [19, 20].

In this context, a 5-HT_6_ PET radiotracer could be an interesting tool for such purpose in obesity. [^18^F]2FNQ1P is the first fluorinated radiotracer with high affinity and selectivity for 5-HT_6_ receptors [21], enabling cerebral 5-HT_6_ receptor changes to be tracked in vivo via radiotracer uptake in the brain. We aimed at exploring the influence of a DIO model and of genetic obesity on 5-HT_6_ receptor density, using the PET radiotracer [^18^F]2FNQ1P [22]. The main goal was to detect a modification of 5-HT_6_ receptor density in Wistar triggered by high-fat diet.

## Methods

### Pre-registration

This study was preregistered [23]. Results of a pilot study, along with justification and deviations from the experimental plan are summarized in the Supplementary.

### Study design, animals and diet

All experiments were carried out under a protocol approved by the local review board (“Comité d’éthique pour l’Expérimentation Animale Neurosciences Lyon”, registration code: C2EA—42), authorized by the French Ministry of Higher Education and Research (no. 25505-2020052514313066V6, and were in accordance with European directives on the protection and use of laboratory animals (Council Directive 2010/63/UE; French decree 2013-118). Animals were housed in standard temperature and humidity conditions with a 12 h/12 h light/dark cycle, with environmental enrichment and 2–5 animals per cage. Four groups of rats were followed up for 10 weeks: (1) male Wistar rats (Crl:WI, Charles River Laboratories^®^; 5–6 weeks old, 175–200 g) fed with high-fat diet (SAFE^®^ U8955 pellets, 246HF), as “Wistar DIO group” (*n* = 13); (2) control male Wistar rats fed with normal diet as “Wistar control diet group” (*n* = 7); (3) male Zucker rats with obesity (Crl:ZUC(Orl)-*Lepr*^*fa*^, Charles River Laboratories^®^; 5–6 weeks old, 175–200 g) fed with normal diet but developing genetic obesity (*n* = 7); and (4) male Zucker lean strain rats (Crl:ZUC-Lepr^*fa*^, Charles River Laboratories^®^; 5–6 weeks old, 175–200 g) fed with normal diet considered as genetic control (*n* = 4). Diet composition and duration were chosen in the light of the literature [24] and were validated together with the rat strain in a pilot study, as described in the preregistration document [23]. Food (and energy) intake was measured by weighing the food each time it was replenished. At the end of the experiment, the total weight of the consumed food was divided by the number of days in the experiment and the number of rats in the cage. Day 1 of the study protocol was defined by the day of starting diet. Weight and food consumption were recorded twice a week. 7T magnetic resonance imaging (MRI) was performed to track whole-body fat increase at baseline and once the animals reached a ceiling of 500 g (after 5 weeks of experiment), beyond which they cannot fit into the MRI scanner. [^18^F]2FNQ1P PET/CT (computed tomography) was performed at baseline and after 10 weeks, to assess 5-HT_6_ density before and after experiment. A flow-chart resuming study design is presented in Supplementary. The study was designed to detect a 20% difference in radiotracer uptake before and after experiment with 10–20% variability, 5% alpha risk and 80% power (primary outcome). The study was not powered for comparisons between Wistar DIO rats, Zucker with obesity, Wistar control diet rats and Zucker lean strain (secondary, exploratory outcomes). A permeability test with Evans blue dye in a subset of three rats each for the DIO, control and Zucker groups was performed to assess blood-brain barrier (BBB) integrity (results shown as Supplementary) [25].

### Radiosynthesis of the 5-HT_6_ receptor radiopharmaceutical and quality controls

The chemical nitro-precursor of our 5-HT_6_ PET radiotracer, [^18^F]2FNQ1P, was synthesized as described previously [22]. Radiolabeling with ^18^F was performed extemporaneously, on the days of experiments, according to our published protocol [21]. Briefly, radiosynthesis used an automated Neptis^®^ fluorination module (ORA Neptis^®^, Belgium). [^18^F]2FNQ1P quality control determined radiochemical purity and molar activity on analytical HPLC assay at the end of each production run, guaranteeing the radiopharmaceutical quality of the radiotracer used for the in vivo experiments: i.e., molar activity >75 GBq/µmol and radiochemical purity >98%.

### Imaging protocol

All imaging sessions were performed under isoflurane anesthesia (induction with 4% and maintenance at 2%), delivered in air by approved systems (TEM Sega). Rectal temperature was continuously measured and maintained at 37 ± 1 °C.

### MRI

The MRI experiments were conducted on a 7T small animal Bruker system (Bruker, Germany) equipped with a 12-cm actively shielded bore and 440 mT/m gradient set. The animals were placed in prone position on a dedicated plastic bed (Bruker Biospec Animal Handling Systems, Germany), adapted with a stereotactic system for immobilization. A respiratory sensor was placed on the animal’s abdomen to constantly monitor respiration. MRI acquisitions were made using a whole-body emission-reception body coil (72 mm). First, a reference scan in 3 directions was acquired to adjust shim and frequency parameters over the entire body and to position the coronal stack for whole-body subcutaneous and visceral fat analysis. For the water image, a 2D T1-weighted respiratory gated spin-echo sequence (MSME) with fat suppression was acquired with the following parameters: TR/TE = 6164/8.1 msec, and matrix size 256 × 128 for voxel size 586 × 625 × 1000 µm^3^. Fat images were acquired using the cloned MSME sequence of the water image with a B1 shift frequency of 1050 Hz corresponding to the water-fat frequency gap at 7T (300 MHz). Total intra-abdominal and subcutaneous fat volume was measured under ImageJ software by binarizing the fat images, and then extracted using a dedicated volume quantification plugin and expressed in cm^3^.

### PET/CT

Hydration with 2 mL physiologic serum i.p. was performed after the start of anesthesia. Tariquidar (8 mg/kg) was administered in the caudal vein to block PgP activity and enhance brain delivery of the radiotracer. Thirty minutes later, [^18^F]2FNQ1P (37 kBq/g) was administered in the caudal vein. Static brain PET acquisitions were then made 40 min after radiotracer injection and for 20 min on a Inveon PET/CT scanner (Siemens), with animals in prone position, head centered in the field of view, and monitoring of respiratory rate. CT scanning was performed to correct attenuation and scatter. Images were reconstructed with attenuation and scatter correction by 3D ordinary Poisson ordered subsets expectation-maximization (OP-OSEM3D) with 4 iterations and a zoom factor of 2. The reconstructed volume was constituted of 159 slices of 128 × 128 voxels, in a bounding box of 49.7 × 49.7 × 126 mm^3^ and with voxel size 0.388 × 0.388 × 0.796 mm^3^. Images were analyzed with the Inveon Research Workplace (IRW, Siemens) for region of interest (ROI) analysis and SPM12 (Wellcome Trust Center for Neuroimaging, London, UK) for voxel-based analysis. The efficiency of tariquidar in enhancing radiotracer delivery to the brain and ability to quantify uptake from static acquisitions were validated on test-retest dynamic scans performed in Wistar rats, as previously reported in a pilot study described in the preregistration document [23].

Uptake, expressed in Bq, was normalized for injected dose (corrected for radioactive decay) and for weight to obtain standardized uptake values (SUV). To obtain a SUV ratio (SUVr), SUV in each voxel was normalized to whole brain SUV. Thus, parametric images of SUVr were obtain at baseline and week 10 after diet. Individual CT images of each time point were realigned and then spatially normalized on a MRI template with automatic and elastic correction. These CT-based transformations were then applied to the corresponding PET images. Two different MRI templates were used: the SIGMA MRI template for Wistar rats and a home-made MRI template, realigned on SIGMA MRI template, for Zucker rats since no specific template has been published yet for this strain. Brain MRI template was obtained from 4 Zucker rats with obesity. A visual check of the template with the Zucker lean strain was performed to validate its use. All images were then visually checked in order to verify the quality of the coregistration.

Parametric images were smoothed using an isotropic Gaussian filter (1.0 × 1.0 × 1.0 mm) and then used for both ROI analysis and voxel-based analysis.

For pre-specified ROI analysis, a limited number of ROIs involved in food intake for which we had an a-priori hypothesis were used to perform multiple comparisons: striatum, hypothalamus, hippocampus, amygdala and frontal cortex. Small ROIs were manually drawn, within each of these regions, on the SIGMA atlas [26] and then used to extract regional SUVr in both Wistar and Zucker rats. A flow-chart has been added in the Supplementary to explain the analysis process in detail.

### Statistical analyses

All results are reported as mean ± standard deviation. Statistical analyses, with the *p* value threshold set at 0.05, were performed as follows to respond to the pre-specified hypotheses. Considering size samples, non-parametric tests were performed. Kruskal–Wallis test with Mann–Whitney bilateral post-hoc tests were performed to assess weight, food consumption and MRI-related changes between groups. The primary outcome (PET changes before and after diet) was assessed with the following analyzes.

1. ROI analysis: Wilcoxon paired comparison tests were performed to assess differences in [^18^F]2FNQ1P uptake before and after experiment (10 weeks). Due to multiple comparison for the 5 regions tested, Bonferroni correction was applied to consider *p* < 0.01 as significant.
2. Voxel-based analysis: SPM12 was used to compare groups longitudinally (baseline vs. 10 weeks) using the statistical parametric mapping approach. Parametric images were compared between the two time-points voxel by voxel on paired *t*-test. The cluster-forming threshold was set at *p* < 0.05 (family-wise error (FWE) corrected). Clusters with an extent of ≥60 voxels (thus matching the raw voxel size of PET) and with *p* < 0.05 FWE corrected were considered significant.

Secondary outcomes were assessed with the following. Kruskal-Wallis test with Mann–Whitney bilateral post hoc tests were performed to assess [^18^F]2FNQ1P binding between the Wistar DIO, Wistar control diet, Zucker with obesity and Zucker lean strain groups.

## Results

### Weight and food consumption

Mean weights and food consumption results are reported in Table 1. Statistical tests showed no differences in mean weights between groups at beginning and end of experiment (*p* > 0.05). Food consumption expressed in quantity of pellets (g/day/rat) showed no differences between groups. Energy absorbed per day in the Wistar DIO group was significantly higher than in the other groups (*p* = 0.0002, with a higher mean ranging of 43%, 67% and 95% compared to Wistar control diet, Zucker with obesity and Zucker lean strain groups, respectively).

**Table 1:** Mean weights before and after experiment and mean food consumption (weight and nutritional content) for the four different groups.

Groups	Starting weight (g)	Final weight (g)	Weight increase (%)	Food consumption (g/day/rat)	Energy intake (kJ/day/rat)
Wistar DIO	272 ± 55	596 ± 75	125 ± 47	22 ± 4	448 ± 79^a^
Wistar control	259 ± 23	560 ± 46	118 ± 32	24 ± 3	314 ± 38
Zucker with obesity	259 ± 51	529 ± 29	110 ± 38	21 ± 2	268 ± 21
Zucker lean strain	269 ± 15	451 ± 16	68 ± 4	18 ± 1	230 ± 13

Results expressed as mean ± SD.

^a^Highlight group significantly different from others (*p* = 0.0002).

### MRI analysis

**Fig. 1:**
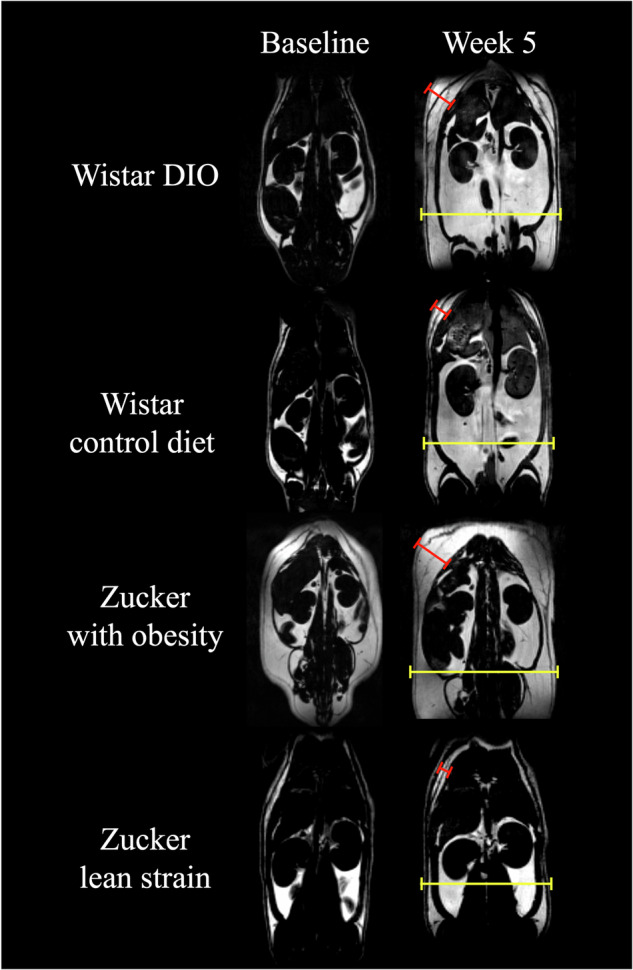
7T MRI images of body-fat gain at baseline and 5 weeks later for each group. Fat volume is represented in white and water in dark. Bars point fat volume in subcutaneous (red) and abdominal (yellow). Subcutaneous bars sizes were 0.78 cm, 0.45 cm, 1.00 cm and 0.34 cm for DIO rat, Wistar control diet, Zucker with obesity and Zucker lean strain, respectively. Abdominal bars sizes were 3.76 cm, 3.50 cm, 3.97 cm and 3.50 cm for DIO rat, Wistar control diet, Zucker with obesity and Zucker lean strain, respectively.

**Fig. 2:**
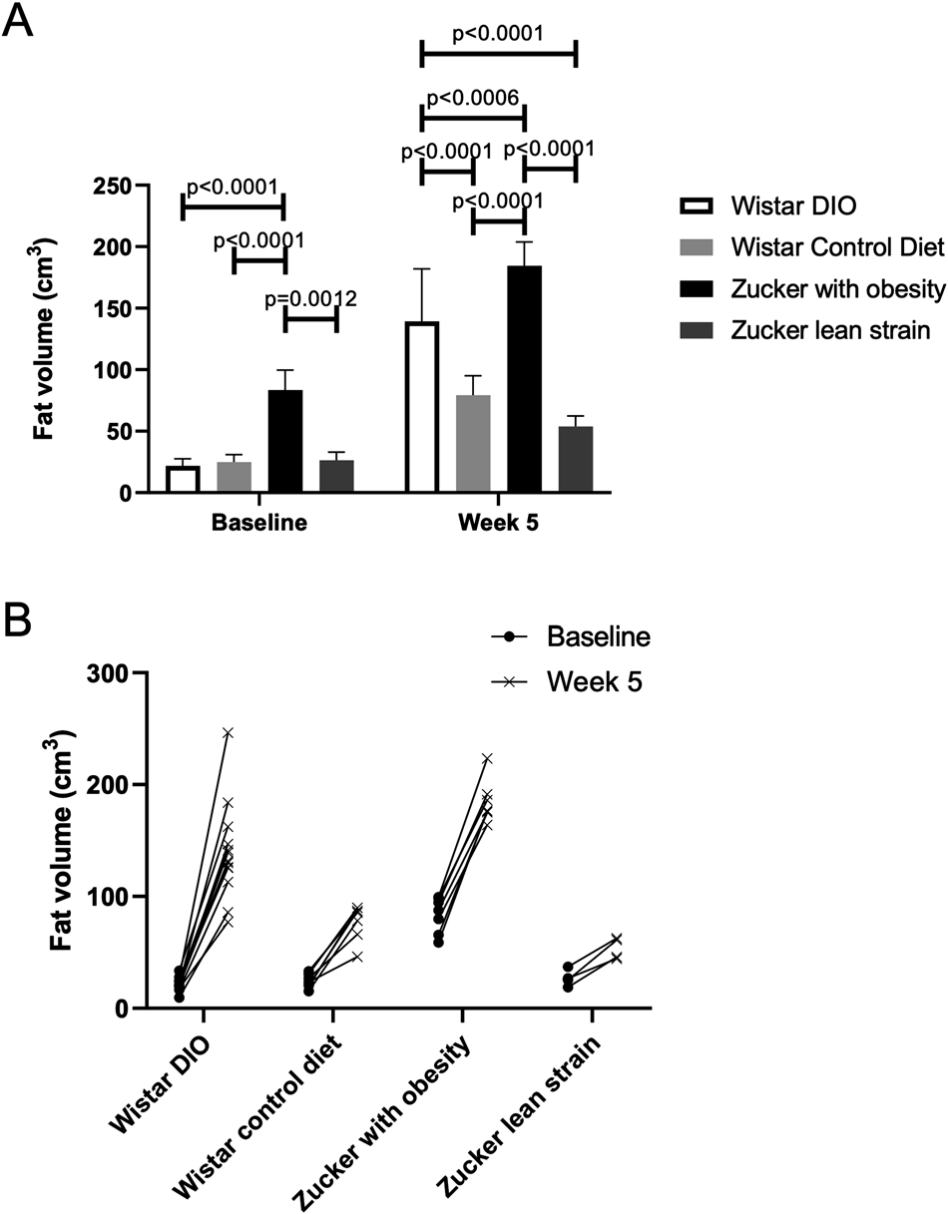
Evolution of fat volume in groups from baseline to week 5. **A** Mean MRI measurements of fat volume (in cm^3^) and comparison between groups at baseline and 5 weeks after experiment. Bar plots express mean ± SD. **B** Evolution per rat of each group of the fat volume between baseline and after 5 weeks of experiment.

MRI was limited to 5 weeks, because, after that time, animals with obesity couldn’t be adequately positioned in the MRI tunnel. Figure 1 shows representative images for each group, highlighting marked subcutaneous and abdominal changes over this 5-week period. Fat volume differences between Wistar DIO group, Wistar control diet group, Zucker rats with obesity and Zucker lean strain rats are summarized in Fig. 2A. Statistical tests showed significant differences *between* groups both at baseline (*H* = 16.87, 3 DF, *p* = 0.0008) and after experiment (*H* = 22.48, 3 DF, *p* < 0.0001). Figure 2B shows the individual evolution of the fat volume over the 5 weeks of experiment. The Wistar DIO group showed the most important variation (*p* < 0.0001), with a 557% ± 160% increase in body fat volume, while Wistar control diet, Zucker with obesity and Zucker lean strain groups showed a 240% ± 120%, 127% ± 41% and 104% ± 43% increase in fat volume, respectively. Results of fat volume quantification is presented in Supplementary Table S1.

### PET analysis

#### Intra-group differences

**Fig. 3:**
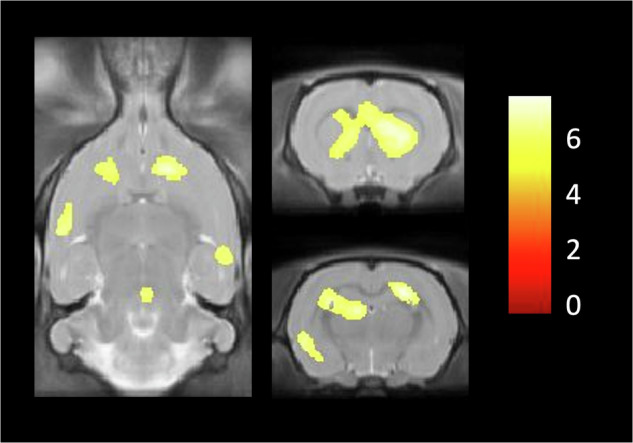
Cluster differences in DIO rats between baseline and after high-fat diet (*p* < 0.05 FWE corrected). Cluster differences are projected on MRI templates. Differences were located in different regions: hippocampus, striatum, cingulate, temporal cortex and brainstem. Color bar represents *Z* score.

Intra-group analysis using voxel-based showed six clusters of uptake differences in the Wistar DIO group (*n* = 13) comparing baseline and 10-week values, spread across different focal sub-regions: hippocampus, striatum, cingulate, temporal cortex and brainstem (*p* < 0.05 FWE corrected, statistics of each cluster are presented in Supplementary Table S3). Cluster volumes of increased 5-HT_6_ receptor density after diet ranged from 2 to 35 mm^3^. Figure 3 shows cluster differences projected onto an MRI template in the Wistar DIO group. The ROI-based analysis partially confirmed these results, by identifying pre/post-diet differences in the DIO group in striatum. Finally, additional exploratory analyses showed no cluster differences in the Wistar control diet (*n* = 7), Zucker with obesity (*n* = 7) and Zucker lean strain (*n* = 4) groups (*p* > 0.05, FWE corrected).

#### Inter-group differences

Mean SUVr values extracted from manual ROI delineation for baseline and after experiment (week 10) are shown in Supplementary Table S2. The results of inter-group comparisons are summarized in Fig. S1. Briefly, Zucker rats with obesity showed higher uptake in hypothalamus (at 10-week) and amygdala (at baseline and 10-week) compared to Wistar DIO group and Wistar control group. Both Zucker rat strains showed lower uptake in striatum compared to Wistar DIO and control group at baseline. At 10-week, only Zucker with obesity showed lower uptake in striatum compared to Wistar DIO and control group. Importantly, these differences were related to small effect size (mean differences <15%).

## Discussion

In this study, we performed longitudinal MRI and PET in 4 experimental groups to assess the influence of high-fat diet and genetic obesity on the density of cerebral 5-HT_6_ receptors. The main finding, based on voxel analysis, was that high-fat diet increased 5-HT_6_ expression in several focal regions: striatum, hippocampus, cingulate, temporal cortex and brainstem.

### Body-fat MRI images to decipher DIO models

MRI was performed to assess body-fat gain and validate the models of DIO and genetic obesity. Interestingly, all groups of rats showed similar weight progression and final weight was not a discriminating factor to assess obesity differences between groups. MRI imaging discriminated groups based on body-fat gain using fat volume measurement. We showed the relevance of MRI-based monitoring of diet-induced obesity, which could be very useful in identifying some rat strains failing to show a DIO pattern when fed with a high-fat diet [27].

### High-fat diet increase 5-HT_6_ expression in brain

In the Wistar DIO group, a 10-week high-fat diet was able to trigger 5-HT_6_ receptor changes in various brain regions: hippocampus, striatum, cingulate, temporal cortex, and brainstem. These results are in accordance with 5-HT_6_ receptor expression in these regions [28, 29] and their involvement in food intake regulation [30]. The serotoninergic signaling pathway in feeding behavior can be divided in two: reward and homeostatic pathways [6].

The hippocampus, striatum, cingulate and temporal cortex are part of the reward system, while the brainstem and especially the raphe is part of the homeostatic pathway. In hippocampus, 5-HT_6_ receptors have been largely described [31]. In preclinical studies, obesity due to high-fat diet, showed a deterioration of hippocampus in rats by affecting hippocampal proteome and neurogenesis [32]. Obesity have been identified as a risk factors for hippocampus impairment which could led to neurodegeneration disease [33]. The striatum is known to express relatively high levels of 5-HT_6_ receptor [10]. It is part of the reward circuit, thought to be involved in food consumption regulation [34] and pharmacological response to 5-HT_6_ agonist in food intake [35]. More precisely, striatum have been shown to be implicated in food craving and weight gain in patients with obesity [36, 37]. The cingulate is well described in the reward system [38]. Anterior cingulate cortex has shown to be involved in food craving. A negative correlation have been shown between anterior cingulate cortex and body mass index during food-inhibition task [39]. Also, anterior cingulate cortex showed hyperactivation to food-cue in patients with binge eating disorders [40]. In posterior cingulate cortex activation during high-calorie food anticipation was highly correlated to body mass index [36]. Finally, in PET studies, temporal cortex showed to be associated with hunger and satiety [41, 42]. This mechanism might implicate 5-HT_6_ receptor since antagonist, and agonist drugs induced satiety [19]. In obesity, gray matter volume was found to be negatively correlated to body mass index in humans [43, 44]. Moreover, preclinical studies showed that alterations of temporal cortex led to hyperphagia and obesity [45].

In the homeostatic system, the brainstem is known to possess a high density of serotonin receptors. Although 5-HT_6_ receptor expression is low in brainstem [46], our results suggest a role of this receptor in these mechanisms. Brainstem can be dived in several nuclei: raphe nuclei, nucleus tractus solitarius and parabrachial nucleus. Raphe nuclei are involved in orexigenic effects by inhibiting serotoninergic projections, while nucleus tractus solitarius and parabrachial nucleus are involved in anorexigenic effects [6]. Overall, to our knowledge, this is the first study to demonstrate 5-HT_6_ alterations resulting from high-fat diet. However, our study does not speculate on the mechanism involved in these 5-HT_6_ modifications. Further mechanistic studies are required to elucidate them.

Interestingly, no significant differences were found in the Wistar control diet group, Zucker rats with obesity or Zucker lean strain rats between baseline and 10-week values. This suggests that the differences in the Wistar DIO group were not due to brain development but specifically to the high-fat diet. Furthermore, the Evans-blue dye test in Wistar DIO group and Wistar control diet group did not show brain permeability, suggesting that there was no disruption of the BBB due to the high-fat diet (see Supplementary).

Comparison between groups, based on ROI analysis, showed contrasted results over brain regions. Overall, Zucker rats with obesity had significant increased uptake in the hypothalamus and amygdala, but decreased uptake in the striatum, when compared to Wistar groups, and, importantly, most comparisons were unaffected by the 10-week high-fat diet. Given the low number of animals enrolled for these exploratory comparisons, we believe that these small differences (in the range of 10–15%) should be interpreted with great caution.

Zucker rats with obesity are deficient for the gene coding for the leptin receptor. Leptin receptor is expressed in the brain, with high levels in the hypothalamus, which is important for regulation of body weight, and leptin binds to the receptor to inhibit food intake [47]. High expression of 5-HT_6_ receptor could then be implicated in obesity development or susceptibility. In support to this idea, hypophagia induced by 5-HT_6_ antagonists has been shown to be mediated by the paraventricular nuclei of the hypothalamus [48]. In the amygdala, the leptin receptor was also reported but to a lesser extent [49, 50] and 5-HT_6_ receptor are expressed in moderate levels [51]. Colocalization of leptin and 5-HT_6_ receptors suggest an interaction between the two. Thus, increases of 5-HT_6_ receptor density in Zucker rats with obesity could be a consequence of the lack of leptin receptors.

Finally, the fact that high-fat diet showed increase 5-HT_6_ density in the striatum while in Zucker rats with genetic obesity showed a decrease is intriguing and suggest a constitutive difference in the transgenic animals which needs to be further explored using animals of different ages.

Several limitations could be considered. Firstly, our results pertain exclusively to male rats. The hormonal cycle in females has a significant impact on neuroplasticity and serotonin production. Furthermore, males are more susceptible to weight gain than females. Additionally, there are gender-based differences in response to a high-fat diet, which could potentially confound our results [52]. Moreover, while interaction between different serotonin receptors (5-HT_2C_, 5-HT_1B_) and leptin remain unclear, further studies are also required to explore the potential interaction with 5-HT_6_ receptors [53]. In this regard, it is important to note that rats were not fasting before PET images, which might influence [^18^F]2FNQ1P distribution. To address this concern, we conducted additional, separate experiments to assess the influence of fasting on radiotracer distribution. Four different rats underwent two randomized PET scans using [^18^F]2FNQ1P, one with a 4 h fasting period before imaging and the other under the same conditions as described in our study (no fasting). The images were analyzed using SPM analysis, as described in our study (with the operator blinded) and did not reveal significant differences suggesting no influence of fasting on radiotracer distribution. Finally, in PET exploration, we were unable to formally differentiate whether the increased binding is likely due to an increase in expression or a decrease in serotonin levels reducing competition with the radiotracer. However, an effect on receptors availability seems unlikely given the high affinity of the radiotracer vs. moderate affinity and concentration of the endogenous serotonin [54, 55].

In conclusion, we performed, for the first time, a PET study in rats, which highlighted the ability to track changes in the density of 5-HT_6_ receptors using the radiotracer [^18^F]2FNQ1P. More generally, brain 5-HT_6_ receptor density could be an interesting biomarker to investigate obesity or eating disorders. Although previous studies reported the implication of 5-HT_6_ in these pathologies, the mechanisms underlying low 5-HT_6_R-mediated signal pathway involved in these functions remain unclear. [^18^F]2FNQ1P could also be an interesting tool to monitor susceptibility for these pathologies to assess the neurological impact of diet on brain changes. The study reinforces the hypothesis that the 5-HT_6_ receptor could be targeted to treat obesity since it showed 5-HT_6_ receptor changes in rats with obesity. Some drugs targeting 5-HT_6_ receptors in obesity have been reported [19, 20] and [^18^F]2FNQ1P could be a reliable tool to assess in-vivo target engagement of the 5-HT_6_ receptor in obesity. Furthermore, new medications arriving on the market such as GLP-1 agonists have been reported to affect serotoninergic system [56, 57]. Further studies using [^18^F]2FNQ1P could investigate potential 5-HT_6_ brain changes under these medications. The challenge will now be to evaluate 5-HT_6_ density in human brain in different population of patients with obesity or eating disorders, to confirm these results.

## Supplementary information

Supplementals

## Data Availability

The datasets generated during and/or analyzed during the current study are available from the corresponding author on reasonable request.
