# Supplementary material for: Perspectives on obesity imaging: [18F]2FNQ1P a specific 5-HT6 brain PET radiotracer
Source: Int J Obes (Lond). 2024 Oct 7;49(1):133–9. doi: 10.1038/s41366-024-01644-x (PMC11683005; doi:10.1038/s41366-024-01644-x)
Supplement: Supplementary file 1 — Supplementals [file 41366_2024_1644_MOESM1_ESM.pdf]

Perspectives on Obesity Imaging: [^{18}F]2FNQ1P a Specific 5-HT$_6$ Brain PET radiotracer

Pierre Courault, Sandrine Bouvard, Caroline Bouillot, Radu Bolbos, Waël Zeinyeh, Thibaut Iecker, François Liger, Thierry Billard, Luc Zimmer, Fabien Chauveau, Sophie Lancelot

Corresponding author: Pierre Courault, pierre.courault@chu-lyon.fr

Supplementals

Content:

1) Methods: preregistration
2) Methods: study design
3) Methods: flow-chart of image preprocessing
4) Table S1: extensive results of MRI fat volume
5) Exploration of BBB integrity using Evans-blue dye method
6) Table S2: extensive results of mean SUVR
7) Figure S1: Results of the ROI-based analysis
8) Table S3: statistics of clusters differences

1. Methods: preregistration

The study was preregistered (https://doi.org/10.5281/zenodo.4700238). In this document, we presented the results of pilot experiments which were helpful to validate several aspects of the study design:

- rat strain (Wistar vs Sprague-Dawley)
- diet (SAFE® U8955, 246 HF), which caused no adverse effects
- MRI protocol (imaging and spectroscopy, both enabling the monitoring of body fat gain)
- use of Tariquidar to enhance brain delivery of $[^{18}F]$2FNQ1P with good repeatability in test-retest experiments (SD < 10% in all brain regions)
- adequate quantification of radiotracer uptake in late, 20-min static scans (rather than dynamic 60-min scans)

Notable deviations from the experimental plan are the following:

- the number of animals included in the main group was lower than planned (13 instead of 18) owing to unexpected difficulties: (i) failure of $[^{18}F]$2FNQ1P radiosynthesis after 10 weeks and impossibility to reschedule led to the loss of 3 rats in the DIO group; (ii) one DIO rat was discarded due to an issue with PET camera during the acquisition on week 10; (iii) one rat died before the end of the experiment
- an additional exploratory group was enrolled in the course of the study: Zucker lean strain rats were used to provide adequate controls for the genetic obesity model

2. Methods: study design

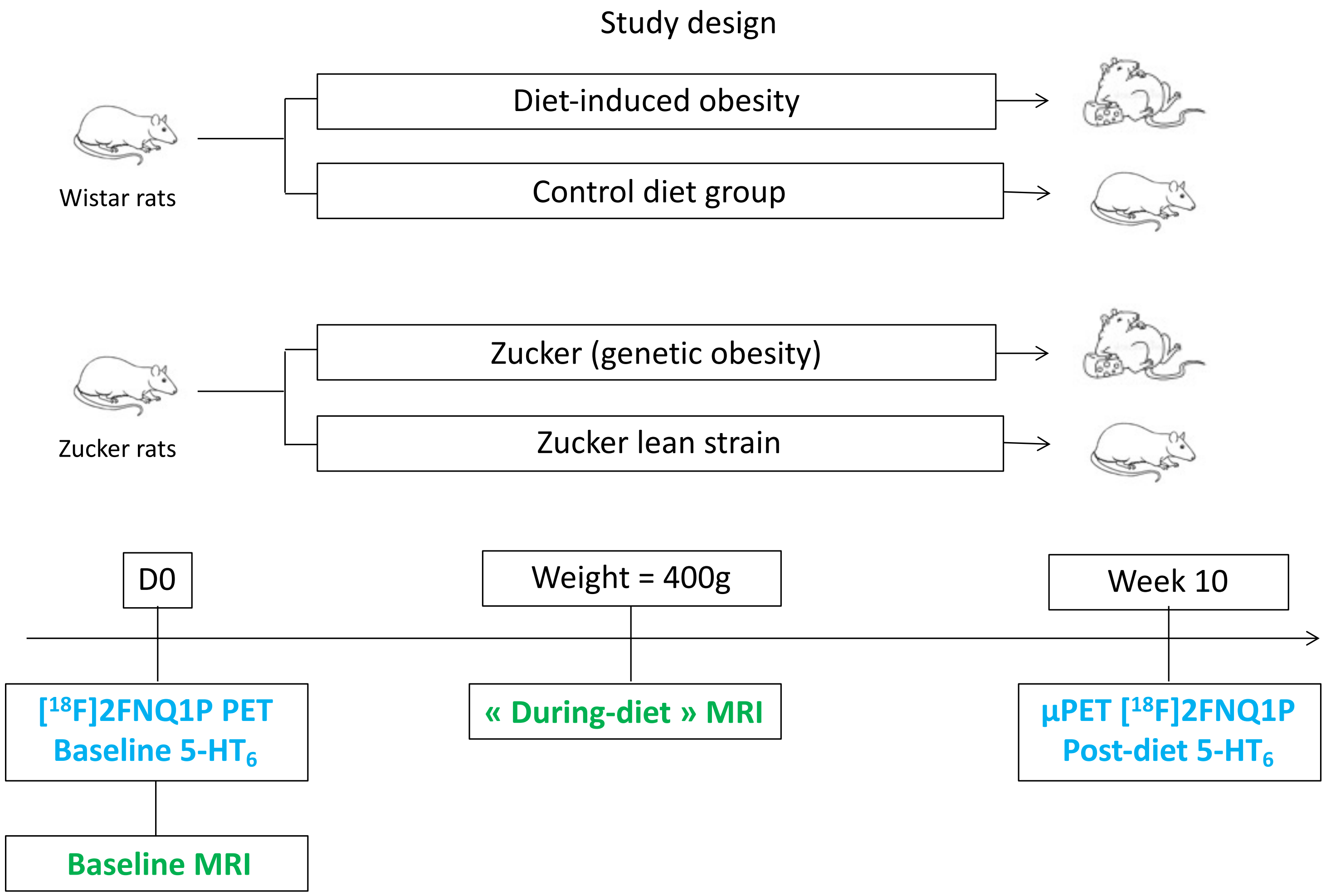

3. Methods: flow-chart of image preprocessing

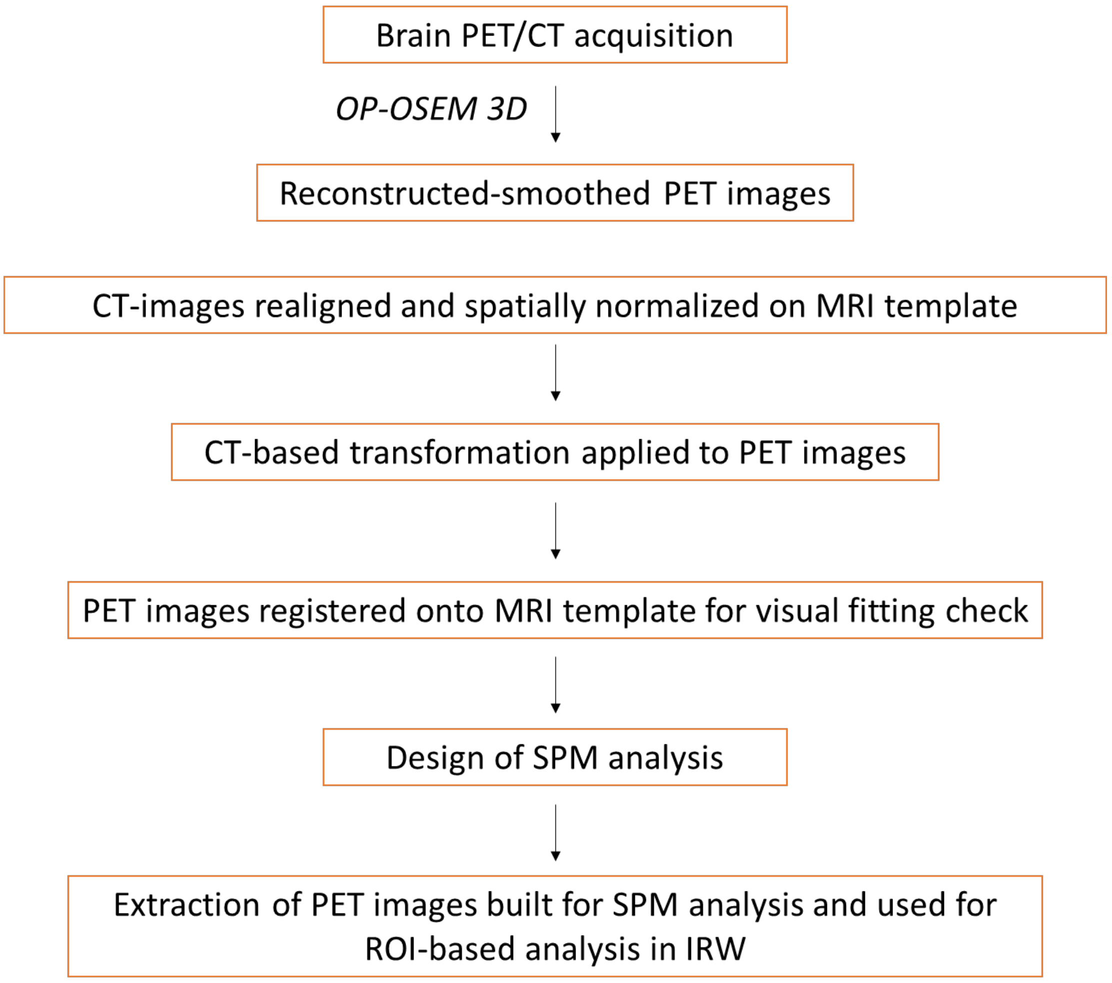

4. Table S1: extensive results of MRI fat volume

MRI measurements for each group at baseline and after 5 weeks of diet. Results expressed as mean ± SD.

	Fat volume (cm^3)	
**Group**	Baseline	Week 5
**Wistar, DIO (n=13)**	21.7 ± 5.9	139.2 ± 42.8
**Wistar, control diet (n=7)**	24.8 ± 6.1	79.3 ± 15.8
**Zucker with obesity (n=7)**	83.5 ± 16.2	184.5 ± 19.3
**Zucker lean strain (n=4)**	26.3 ± 6.7	53.9 ± 8.4

5. Exploration of BBB integrity using Evans-blue dye method

Method

A permeability test with Evans-blue dye in three rats for DIO, control and Zucker group will be performed to assess blood-brain barrier (BBB) integrity. Briefly, rats were anesthetized with isoflurane 2% and a 2% Evans-blue dye solution was administered in caudal vein (4ml/kg). After 20 minutes distribution of dye, rats were perfused transcardially with 250 ml of 0.9% saline for 15 minutes. Rats were then decapitated, and brain was carefully collected and sliced to assess Evans-blue dye brain uptake.

Results

Figure 1 showed brain slice of Evans-blue dye brain uptake for DIO (A), control (B) and Zucker with obesity (C). No Evans-blue dye penetration was visually detectable in DIO and control group. Zucker with obesity rats showed mixed results, one rat out of three showed important Evans-blue dye brain uptake and second without any penetration.

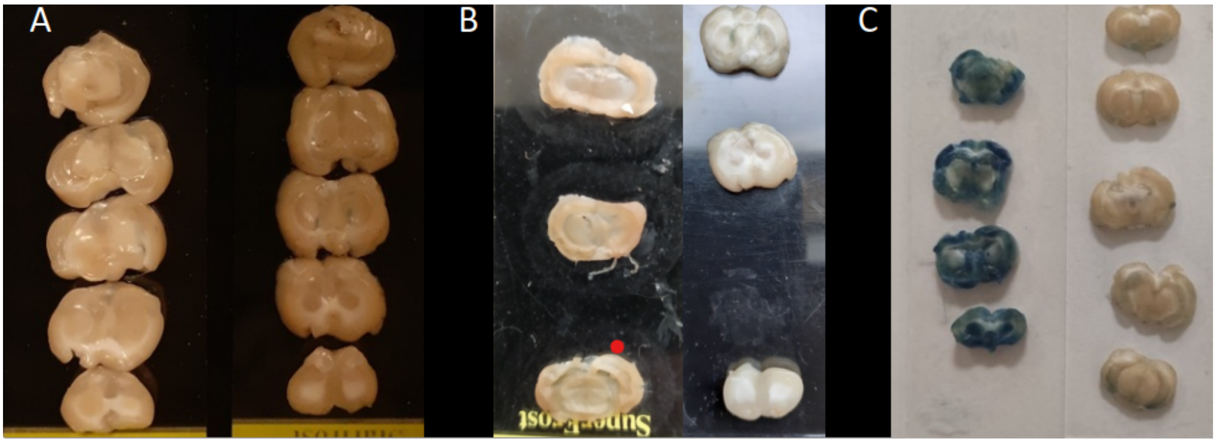

6. **Table S2.** Mean SUVr for each group after manual delineation of ROI in 5 target regions: frontal cortex (FC), hippocampus (Hip), hypothalamus (Hyp), amygdala (Amy) and striatum (Stri). In Wistar DIO group, only striatum showed significant differences between before and after diet (* p-value < 0.05). Whole brain was used as reference region. Results expressed as mean ± SD.

	**Wistar, DIO**		**Wistar, control diet**		**Zucker with obesity**		**Zucker lean strain**	
**Region**	Baseline	Week 10	Baseline	Week 10	Baseline	Week 10	Baseline	Week 10
**FC**	0.99 ± 0.05	1.00 ± 0.08	1.00 ± 0.05	0.98 ± 0.05	1.02 ± 0.03	0.97 ± 0.05	0;97 ± 0.05	1.04 ± 0.04
**Hip**	1.10 ± 0.03	1.17 ± 0.04	1.11 ± 0.03	1.16 ± 0.04	1.07 ± 0.02	1.13 ± 0.09	1.07 ± 0.01	1.18 ± 0.4
**Hyp**	0.95 ± 0.04	0.94 ± 0.06	0.94 ± 0.05	0.93 ± 0.06	1.01 ± 0.04	1.05 ± 0.09	1.02 ± 0.10	0.97 ± 0.03
**Amy**	1.15 ± 0.04	1.14 ± 0.06	1.12 ± 0.04	1.14 ± 0.04	1.29 ± 0.05	1.25 ± 0.15	1.18 ± 0.13	1.16 ± 0.07
**Stri**	1.14 ± 0.03	1.22 ± 0.03	1.16 ± 0.05	1.23 ± 0.03	1.03 ± 0.04	1.07 ± 0.09	1.01 ± 0.06	1.15 ± 0.06

7. **Figure S1:** Results of the ROI-based analysis. Mean SUVr per region for Wistar DIO (n=13), Wistar control diet (n=7), Zucker with obesity (n=7) and Zucker lean strain (n=4) groups at baseline and end of experiment (10 weeks) in striatum, amygdala and hypothalamus. Results expressed as mean ± SD (** p < 0.01 ; **** p < 0.0001).

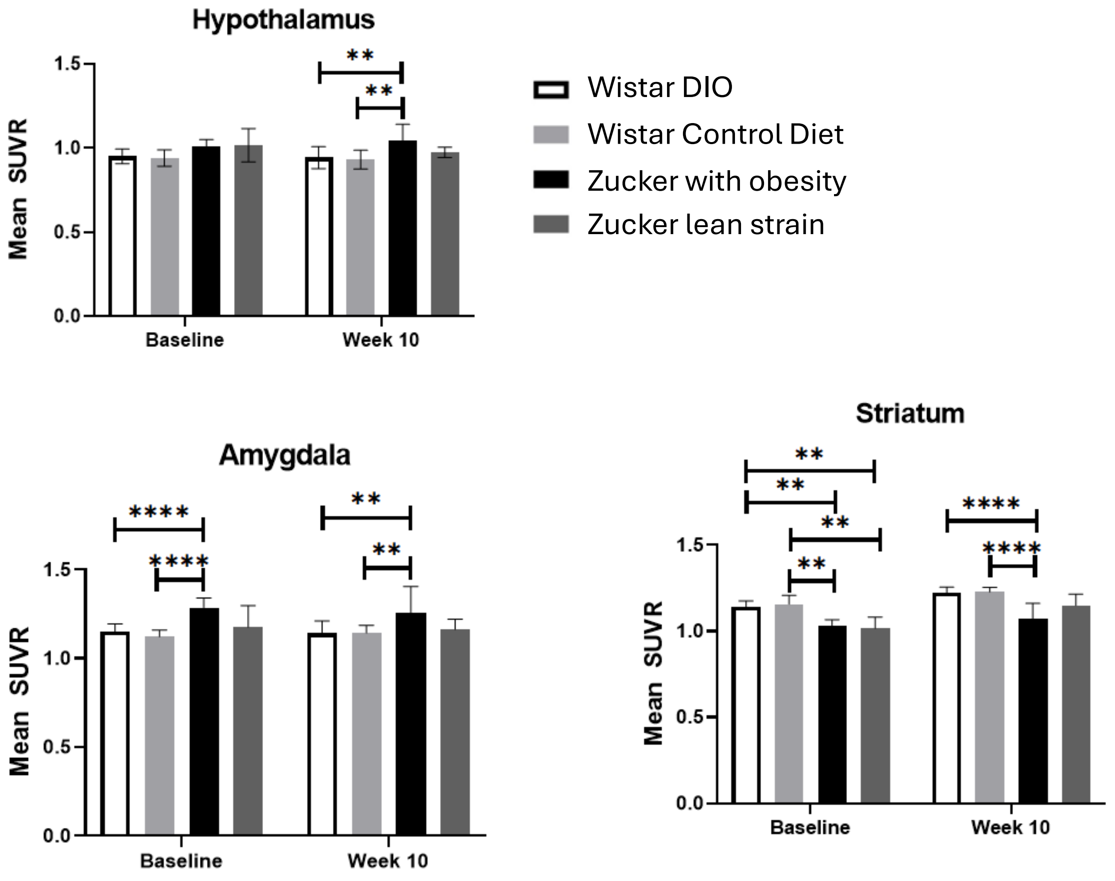

8. Table S3. Statistics of clusters differences.

	Cluster-level			Peak-level		
**Cluster number**	p-value (FWE-corrected)	Size (Number of voxels)	Regions included in the cluster	p-value (FWE-corrected)	T value	Z_E
**1**	< 0.0001	5318	Hippocampus	0.003	7.56	5.01
**2**	< 0.0001	16501	Striatum, Cingulate, Hippocampus	0.005	7.24	4.89
**3**	< 0.0001	8123	Striatum, Hippocampus	0.008	6.84	4.74
**4**	< 0.0001	2801	Frontal cortex	0.010	6.70	4.69
**5**	0.001	1390	Temporal cortex	0.017	6.40	4.56
**6**	0.012	293	Brainstem	0.018	6.35	4.54
